# Cellular Changes during Renal Failure-Induced Inflammatory Aortic Valve Disease

**DOI:** 10.1371/journal.pone.0129725

**Published:** 2015-06-12

**Authors:** Mony Shuvy, Suzan Abedat, Mahmoud Mustafa, Nitsan Duvdevan, Karen Meir, Ronen Beeri, Chaim Lotan

**Affiliations:** 1 Schulich Heart Centre, Sunnybrook Health Sciences Centre, Toronto, Ontario, Canada; 2 Cardiovascular Research Center, Hadassah-Hebrew University Medical Center, Jerusalem, Israel; 3 Department of Pathology, Hadassah-Hebrew University Medical Center, Jerusalem, Israel; Brigham and Women's Hospital, Harvard Medical School, UNITED STATES

## Abstract

**Background:**

Aortic valve calcification (AVC) secondary to renal failure (RF) is an inflammation-regulated process, but its pathogenesis remains unknown. We sought to assess the cellular processes that are involved in the early phases of aortic valve disease using a unique animal model of RF-associated AVC.

**Methods:**

Aortic valves were obtained from rats that were fed a uremia-inducing diet exclusively for 2, 3, 4, 5, and 6 weeks as well as from controls. Pathological examination of the valves included histological characterization, von Kossa staining, and antigen expression analyses.

**Results:**

After 2 weeks, we noted a significant increase in urea and creatinine levels, reflecting RF. RF parameters exacerbated until the Week 5 and plateaued. Whereas no histological changes or calcification was observed in the valves of any study group, macrophage accumulation became apparent as early as 2 weeks after the diet was started and rose after 3 weeks. By western blot, osteoblast markers were expressed after 2 weeks on the diet and decreased after 6 weeks. Collagen 3 was up-regulated after 3 weeks, plateauing at 4 weeks, whereas collagen 1 levels peaked at 2 and 4 weeks. Fibronectin levels increased gradually until Week 5 and decreased at 6 weeks. We observed early activation of the ERK pathway, whereas other pathways remained unchanged.

**Conclusions:**

We concluded that RF induces dramatic changes at the cellular level, including macrophage accumulation, activation of cell signaling pathway and extracellular matrix modification. These changes precede valve calcification and may increase propensity for calcification, and have to be investigated further.

## Background

Calcific aortic valve stenosis is the most common indication for surgical valve replacement, but there is no effective medical treatment to reduce or slow calcification [1].

The cellular mechanisms of the pathogenesis of aortic valve calcification (AVC) are poorly understood. Yet, in the past decade, studies have demonstrated that AVC is an active, regulated process that involves inflammation [2], alterations in the extracellular matrix (ECM), and differentiation of normal myofibroblasts into bone-producing cells—ie,osteoblasts [3],[4]. These processes contribute to the formation of bone and the calcification of valve leaflets, eventually resulting in valve stenosis.

A significant risk factor for AVC is renal failure (RF). Patients with RF experience more severe calcification, and the rate of calcification is faster compared with patients without RF [5]. To examine the pathogenesis of aortic valve disease—particularly RF-associated valve disease—we developed an animal model based on a unique uremia-induced diet. We have demonstrated that an adenine- and phosphate-enriched diet induces AVC in rats [6], [7]. This unique model is based on the development of transient uremia and mimics the metabolic and hormonal changes that occur in calcification secondary to RF.

We have reported that AVC is an active process that involves inflammation and is associated with an osteoblastic phenotype in valve tissue [6]. Most studies on AVC have examined the endpoint of calcification by comparing calcified with normal valves; however, there are limited data regarding the early phases that lead to calcification.

In this study, we examined the early alterations in the valve before calcification is apparent, hypothesizing that several processes and pathways are activated and suppressed during the course of the disease. Our animal model allowed us to assess various factors and processes, such as inflammation, ECM, protein expression, and osteoblast transformation.

## Methods

### Animal model

Fifty Sprague-Dawley female rats, aged 8 weeks and each weighing approixmately 250 g, were used. The study protocol was approved by the Committee on the Ethics of Animal Experiments of the University of Hebrew University Ethics Committee (Permit Number: MD-12-13407-4). Exsanguination was performed under sodium pentobarbital anesthesia, and all efforts were made to minimize suffering.

The animal model for RF-induced aortic valve disease entailed feeding the rats a high-adenine (0.75%) and high-phosphate (1.5%) diet (Teklad, Madison, WI, USA) [6]. Adenine causes crystal to precipitate in the renal tubules and form 2,8-dihydroxyadenine aggregates [8]. To reproduce this state of abnormal phosphate metabolism in the RF population, phosphate was added to an adenine diet at high concentrations to promote hyperphosphatemia and secondary hyperparathyroidism.

### Study protocol

To focus on the earlier stages of calcification, the experimental design comprised of administering a uremic diet exclusively to rats for various durations.

Fifty rats were fed a phosphate-enriched, uremia-inducing diet for 2, 3, 4, 5, or 6 weeks (10 rats per period). An additional 10 rats served as controls and were fed normal chow.

After each period, the rats were sacrificed by exsanguination and thoracotomy, and the valves were examined by histology, western blot, and immunohistochemistry.

### Effect of diet on biochemical profile

Plasma was analyzed for potassium, phosphate, creatinine, sodium, urea, calcium, and total cholesterol on a VITROS5.1 (Ortho-Clinical Diagnostics, Johnson & Johnson, Rochester, NY).

### Tissue Analysis

Aortic valves were dissected, fixed in formalin, and embedded in paraffin. Serial cross-sections of valve tissue were stained with hematoxylin, eosin, and von Kossa stain.

### Immunoflorescence

Aortic valves (n = 3) were dissected, fixed in formalin, and embedded in paraffin, and 5-μm cross-sections were examined by immunofluorescence. Paraffin was removed with standard xylene washes, and the slides were boiled in 20 mM citrate buffer (pH 6) for 3 min in a pressure cooker to unmask the antigens. To prevent nonspecific binding, the sections were blocked with 3% BSA and 0.1% Tween in PBS for 1 h at room tempreture.

The sections were incubated overnight with anti-collagen 3, anti-collagen 1, anti-fibronectin (Santa Cruz Biotechnology Inc.), anti-CD68 (MP Biomedical), anti-osteopontin (Abcam, Cambridge, UK), and anti-osteocalcin (Santa Cruz Biotechnology Inc.) at 4C. Negative control sections were incubated without primary antibody under otherwise identical conditions. After being washed in PBS, the sections were incubated with Cy5-conjugated secondary antibody (Jackson Immunoresearch Lab; 1:200 dilution) for 1 h.

### Western Blot

Aortic valves from all groups were lysed (n = 3 in each group) in at least two differnt sets. The tissue was hydrolyzed using Hepes buffer (10Mm Hepes,137mM NaCL,4.6Mm KCL,1.1Mm KH2PO4, 0.6Mm MgSO4, 0.1% EDTA, 0.01% digitonin, 1%SDS) with addition of 10 μm protease inhibitor cocktail and homogenized under ultrasound and then boiled for 5 min. Protein concentrations were measured by Bradford assay. Extracts (20 μg) were loaded onto 5% to 15% SDS-polyacrylamide gels and transferred to membranes, which were blotted overnight for Runx-2, osteopontin (Abcam, Cambridge, UK), osteocalcin (Santa Cruz Biotechnology Inc.), ERK, Akt, JNK, P38, (Cell Signaling). Protein levels were normalized to β-actin. The original western blot gels are provided in S1 Fig.

### Image Analysis

Image Pro-Plus version 7.0 (Media Cybernetics, Rock- ville, MD) system was used for image analysis. Twenty random areas from different parts of the valves were analysed by the software. The colors correspondent to positive staining was recorded and numbers of pixels per area and automatic counted on all pictures by the software. For the Western blot we used the Image J software for measurement of bands intensity. All results were normalized to respective controls, which were assigned a nominal value of 1.

### Statistical Analysis

Data were presented as means ± Standard error. Statistical analysis for biochemical profile was analyzed by ANOVA followed by Student-Neumann-Keuls test. P<0.05 were considered significant. Statistical analysis for immunostaining was done using Kruskal-Wallis Test to compare all the groups together, then the non parametric Mann-Whitney Test was used to compare each groups to the control, P<0.05 was considered statistically significant. The pairwise comparisons were carried out using Mann-Whitney Test with Bonferroni correction of the significance level, P< = 0.005 is statistically significant according to the Bonferroni correction.

Statistical analysis for Western blot was done using Wilcoxon Signed Ranks Test. P<0.05 was considered statistically significant.

## Results

### Biochemical profile

After 2 weeks on the adenine diet, the rats experienced a significant increase in creatinine and urea, and after 3 weeks, phosphate levels rose versus the control; RF exacerbated in the Week 5 group and remained unchanged Week 6. In association with RF, the rats developed hypocalcemia after 3 weeks which remained unchanged later. There were no significant changes in potassium, sodium, or cholesterol levels (Table 1).

**Table 1:** Biochemical profile from rats at 2, 3, 5, and 6 weeks.

	Control	Week 2	Week 3	Week 5	Week 6
**Creatinine (μM)**	38±1.5	95±12*	108 ±13*	202 ±41*	213±32 *
**Urea (mM)**	5.5±0.6	17±1.3*	17.9 ±1.7*	36.6± 9*	46.6±10*
**Phosphate (mM)**	2.25±0.1	2.3±0.1	4.07 ±0.7*	6.67± 0.8*	6.09±1.1 *
**Calcium (mM)**	2.39±0.1	2.3±0.1	2.05 ±0.1*	1.88 ±0.3*	1.88±0.3*
**Potassium (mM)**	3.74±0.2	3.1±0.4	3.83±0.5	4.17±0.8	4.07±0.3
**Sodium (mM)**	145±1.5	147±1.6	147±1.6	147±2.2	149±4.7
**Cholesterol (mM)**	2.3±0.2	3.1±0.2	2.56±0.2	2.35±0.3	2.13±0.4

After 2 weeks, there was a significant increase in urea and creatinine levels, reflecting renal failure (RF). After 3 weeks, phosphate levels also increased compared with control; RF worsened in Week 5 and plateaued. As part RF, hypocalcaemia developed after 3 weeks, remaining constant. There were no significant changes in potassium, sodium, or cholesterol levels.

*P < 0.05 between diet groups and control group.

### Histopathology and von Kossa staining

**Fig 1:**
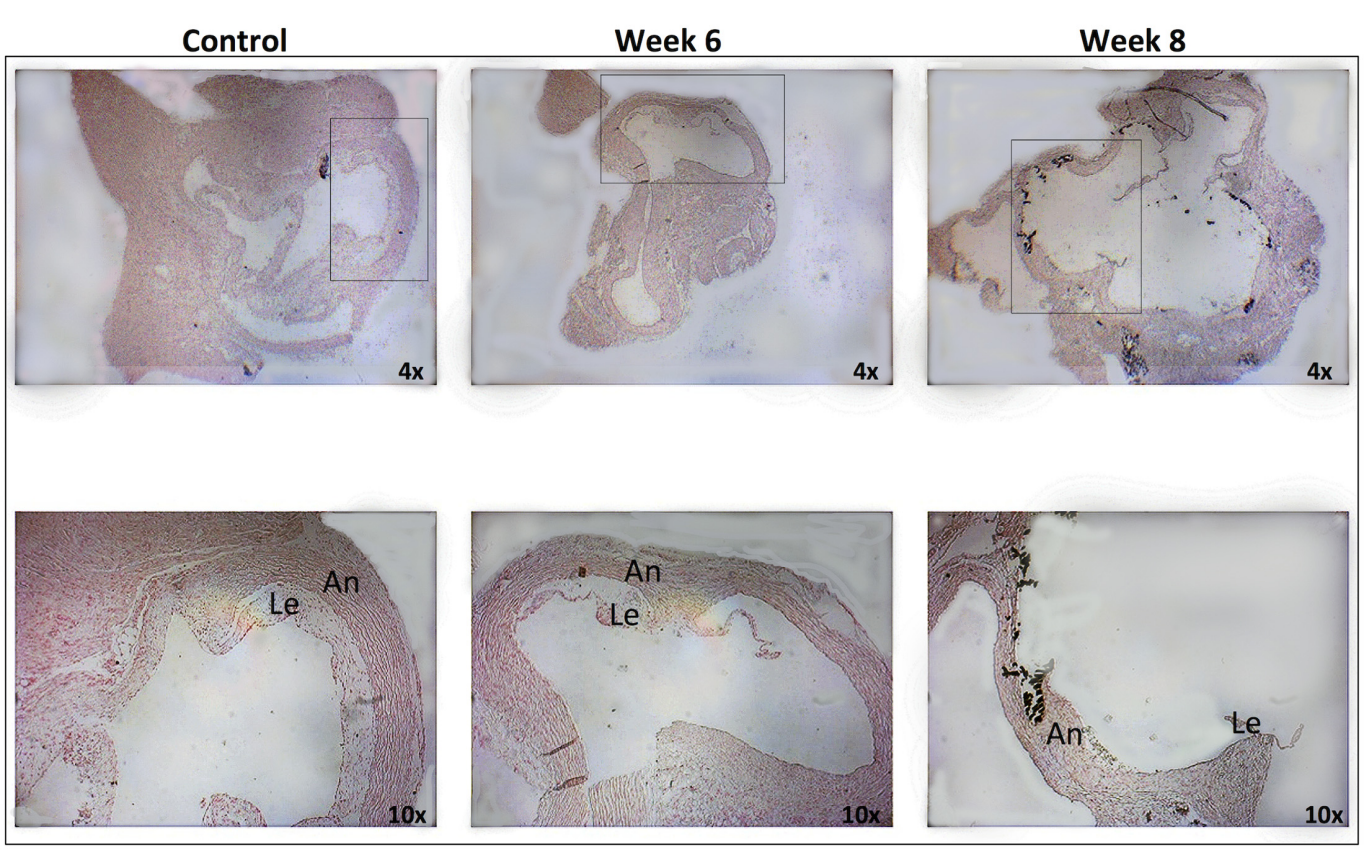
Aortic valve histology. Cross-sections of valves after 6 and 8 weeks on the diet and from controls, showing calcification of the annulus (An) and base of the leaflet (Le) after 8 weeks. No significant calcification was observed in the other groups (von Kossa stain).

Previously, We have demonstrated that rats developed significant valve calcification after 7 weeks on the uremic diet and an additional 2 weeks on a normal diet (Fig 1).

In contrast, by histology, the aortic root sections were unremarkable compared with controls after 2 to 6 weeks on the high-adenine diet. The aortic root sections harbored arterial and valvular tissues, which were stained with hematoxylin, eosin, and von Kossa stain, and failed to show evidence of mineralization (data not shown).

### Inflammation

**Fig 2:**
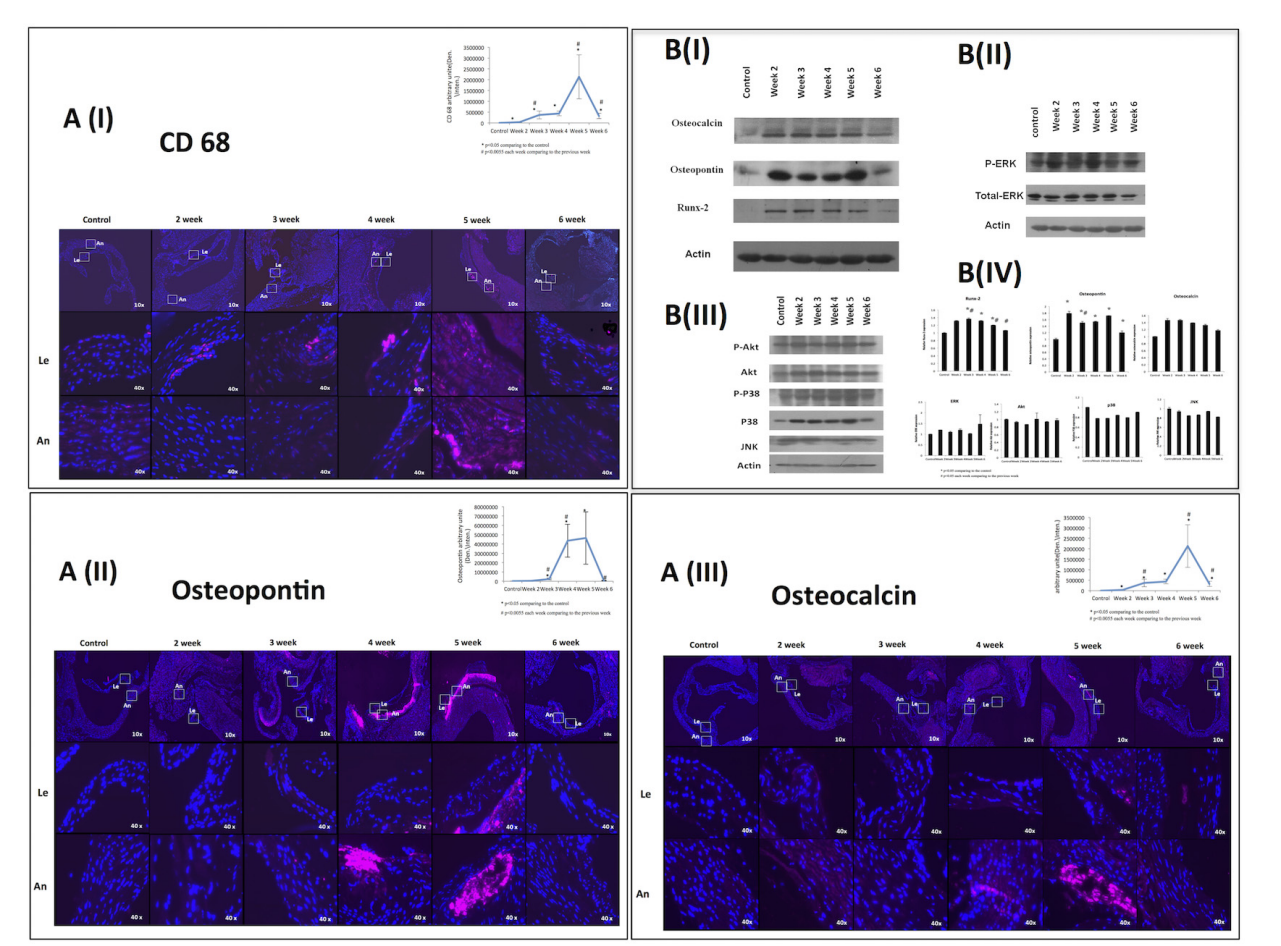
Macrophages osteoblast markers and intracellular pathways in early phases of calcification. (A) Immunostaining of CD68 (I), osteopontin (II), and osteocalcin (III) were positive in the valve annulus (An) and leaflets (Le) after 2 weeks on the nephropathic diet. (B) Western blot analysis (n = 3 in each group) of osteocalcin, osteopontin, and Runx-2 (I), and of the ratio of phosphorylated ERK to ERK-1(II). There were no changes in the expression Akt, JNK, or p38 pathways (III). Graphic presentation of Western blot analysis (IV).

Because AVC involves significant inflammation, we determined the macrophage content in the valves early in the course of the disease by staining for CD68. Macrophages were increased early 2 weeks after the adenine diet was started. Macrophages numbers rose significantly until 5 weeks and were observed in the valve annulus and leaflets. Interestingly, macrophages levels dropped at Week 6 although still remained significantly higher than the controls (Fig 2A(I)). There were no differences in total valvular cells numbers.

### Osteoblast markers

Immunostaining results confirmed the cytoplasmic expression of osteopontin and osteocalcin in valvular cells after 2 weeks as well as in later weeks; their level peaked at 5 weeks and dropped at 6 weeks (Fig 2A(II) and 2A(III)). By western blot, osteopontin peaked at 2 weeks and decreased slightly at 3 and 4 weeks, repeaking at 5 weeks. Like Runx-2, osteopontin expression fell at 6 weeks (Fig 2B (I and VI)).

Osteocalcin was expressed from Weeks 2 to 5, declining in Week 6, although these changes did not reach a statistical significance (Fig 2B (I and IV)).

### MAPK and Akt pathways

**Fig 3:**
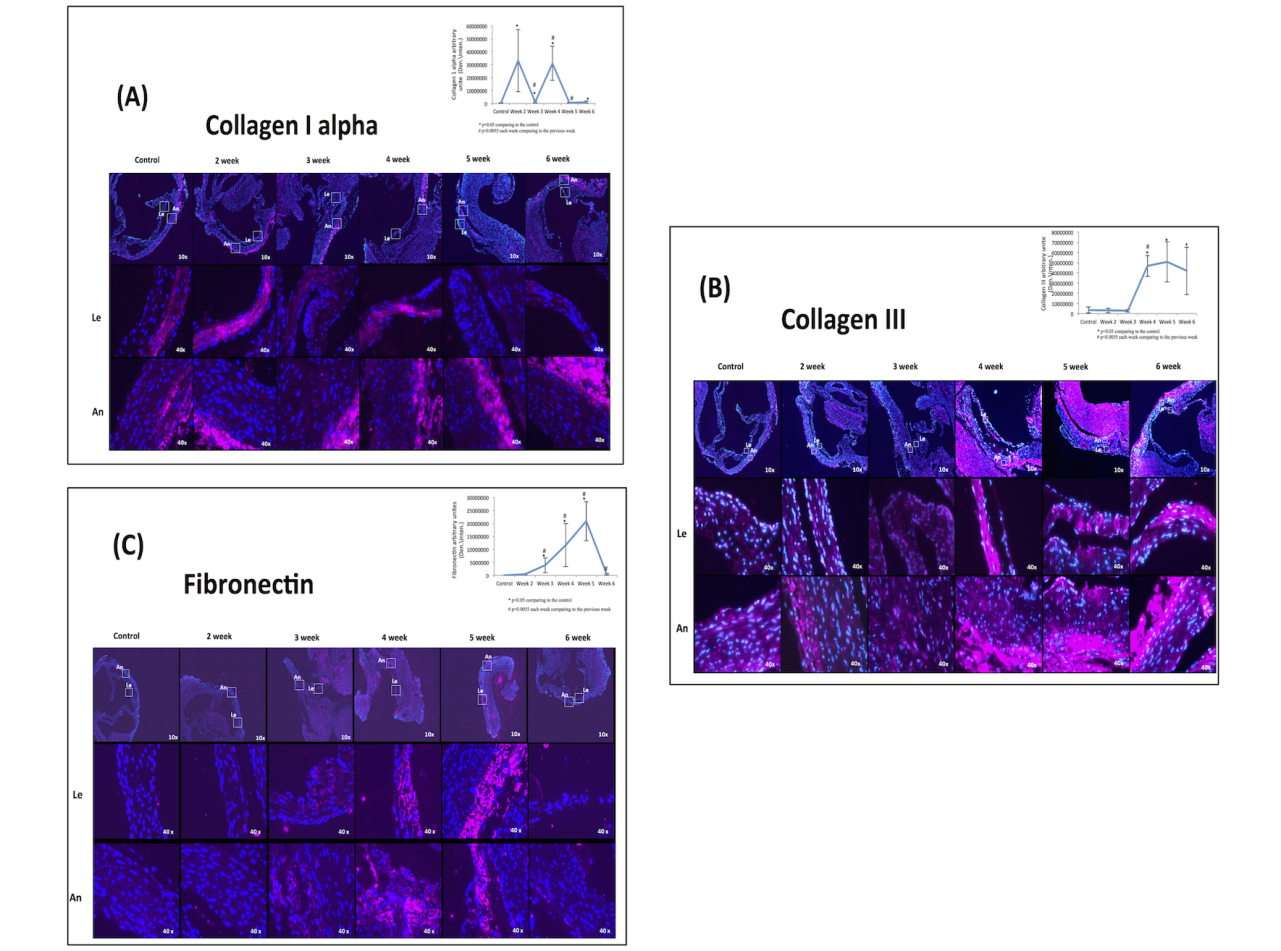
ECM expressions. Aortic valve sections, including the annulus (An) and a leaflet (Le), from study groups before and after immunohistochemical staining. (A) Collagen 1 staining. (B) Staining of collagen 3. (C) Staining of fibronectin.

We evaluated the MAPK and Akt pathways to determine whether they are involved in the early phases of calcification. By western blot, the ratio of phospho-ERK to ERK-1 tended to increase after 2 weeks, fell slightly at 3 and 4 weeks and rose again at 5 weeks (Fig 2B(II)). There were no changes in the expression of Akt, JNK, or p38 pathways (Fig 3B (III and IV)).

### ECM alterations

Collagen 1 levels had 2 peaks of expression at 2 and 4 weeks (Fig 3A). Collagen 3 was expressed after 3 weeks of diet, plateauing at 4,5 and 6 weeks (Fig 3B). Fibronectin levels increased gradually until Week 5 and decreased at 6 weeks (Fig 3C).

## Discussion

Aortic valve disease is a complex disease that involves inflammation, changes in the ECM, and osteoblast transformation in valve tissue. There is no therapy that halts disease progression. Although certain medical therapies, such as statins, might be helpful [9], [10], large clinical trials have contradicted these findings [11],[12]. AVC shares traits with atherosclerosis, such as risk factors [13],[14] and pathological features, but they differ in their response to medical treatment. Whereas atherosclerotic lesions are attenuated and partially reversed with certain treatments, the progression of AVC is intransigent.

The term "aortic valve disease" denotes a wide spectrum of histopathological and clinical situations. In the early stages of the disease, its inflammatory features and extracellular matrix synthesis are more prominent, whereas calcification and bone formation predominate later on [15]. Moreover, therapies that are initiated when the valve is already calcified are ineffective, underscoring the importance of examining the disease in its early stages.

Thus, we evaluated the early phases of the disease—before the valve has been calcified. We used our animal model of RF-associated aortic valve disease, in which calcification is apparent in the valve after 7–8 weeks on a uremia-induced diet.

In this study, we examined valves as early as 2 weeks on the diet until 1 week before the valve became calcified, observing that various osteoblast markers are expressed early in the calcification process, peaking 2 weeks later. Notably, osteoblast marker levels declined before calcification occurred, suggesting that certain processes that modulate calcification are curtailed before it is apparent. Further, the ERK intracellular pathway that was shown previously to be down regulated in calcified valves was upregulated in several points during the inflamatory phase of aortic valve disease.

We also measured several ECM proteins that mediate calcification, noting that ECM protein expression is also an early process that occurs before histological alterations take place and that it entails the modulation of ECM proteins.

### Osteoblastogenesis in AVC

The association between osteogenesis and mineralization of the aortic valve has been well documented in different models over the last decade. Those studies have suggested that mature bone appears in the valve and that cardiovascular calcification resembles bone formation [16],[17]. Further, bone-related proteins are expressed in valvular tissue [18]. Osteoblast markers, such as Runx-2, osteopontin, and osteocalcin are expressed in calcified valves—particularly in calcified areas in the valve [19]. However, the kinetics of calcification and of osteoblast expression have not been examined.

Is this study, we have demonstrated that osteoblast markers are expressed early in the course of the disease—Runx-2, a major transcription factor, and osteocalcin, a marker of mature osteocytes, increased after 2 weeks, remaining highly expressed until Week 5. Osteopontin expression had a unique pattern, peaking at 2 and 5 weeks, which is consistent with data that suggest that osteopontin is involved in several phases of ostoblast maturation process and that its expression peaks twice—early and late [20].

All osteoblast markers were downregulated before calcification was noted in the valve, suggesting that the early active inflammatory process is extinguished before the valve is calcified. This observation might explain the lack of efficacy of therapies, such as statins, that target the inflammatory process, which is absent when the valve has already been calcified.

### ECM in AVC

Several studies have examined the function of ECM in AVC, comparing normal valves with diseased and deformed calcified valves [20],[21]. Whereas collagen types 1 and 3 are present in all 3 layers in normal valves [22], fibrosis is observed in diseased valves, with collagen fibers in the fibrosa becoming disorganized [23]. Collagen and ECM disarray are attributable to increased remodeling that is mediated by elevated activity of matrix metalloproteinases, which mediate the destruction of aortic valvular ECM [24],[25], promoting the progression of aortic stenosis [26]. However, no study has examined the function of ECM in the early phases of the disease before histological changes occur in the valve.

One of the determinants of the cellular phenotype is the ECM [27],[28]; thus, certain ECM proteins have been proposed to influence ectopic calcification by modulating osteoblast transformation. Collagen 1 and fibronectin might promote osteoblast transformation, whereas collagen 4 inhibits it [29]. Notably, knocking out ECM proteins, such as matrix GLA protein, leads to spontaneous formation of ectopic apatite [30]. In this study, alterations in the ECM occurred before histological changes were observed, highlighting their function in inflammation and osteoblast transformation.

## Conclusions

The inflammatory component of the calcification process, which involves macrophage accumulation, activation of intracellular pathways, alterations to the ECM, and osteoblastic features, occurs before calcification appears in the valve. Further, this active process is dowregulated before the valve is calcified. The reasons for down regulation of inflammation are not clear and should be further investigated, a possible explanation is increased apoptosis of inflammatory cells and decreased phagocytosis of apoptotic bodies may accelerate and promote calcification. Importantly, in this study we focused on the inflammatory component of aortic valve disease. Other mediators may be involved in later phases of the process and during the calcification itself. Our results suggest that many processes and pathways are activated and suppressed during the course of the disease. These findings reflect the complexity of the pathogenesis of AVC: various phases might respond differently to medical interventions—eg, a specific therapy that is effective at one stage of the disease might lack efficacy, perhaps causing harm, at other stages.

Additional studies are required for defining the specific phases of calcification as well as the mechanism involved in each phase.

Markers for disease progression must be identified, particularly in patients with RF. Due to the rapid progression of aortic valve disease in this population, a relatively narrow window exists during which medical intervention might be effective.

## Supporting Information

S1 Fig Original Western Blot gels. (PDF)
