# Supplementary figures and images for: Cellular Changes during Renal Failure-Induced Inflammatory Aortic Valve Disease
Source: PLoS One. 2015 Jun 12;10(6):e0129725. doi: 10.1371/journal.pone.0129725 (PMC4466485; doi:10.1371/journal.pone.0129725)

Supplementary Figure 1- Original Western Blot Gels

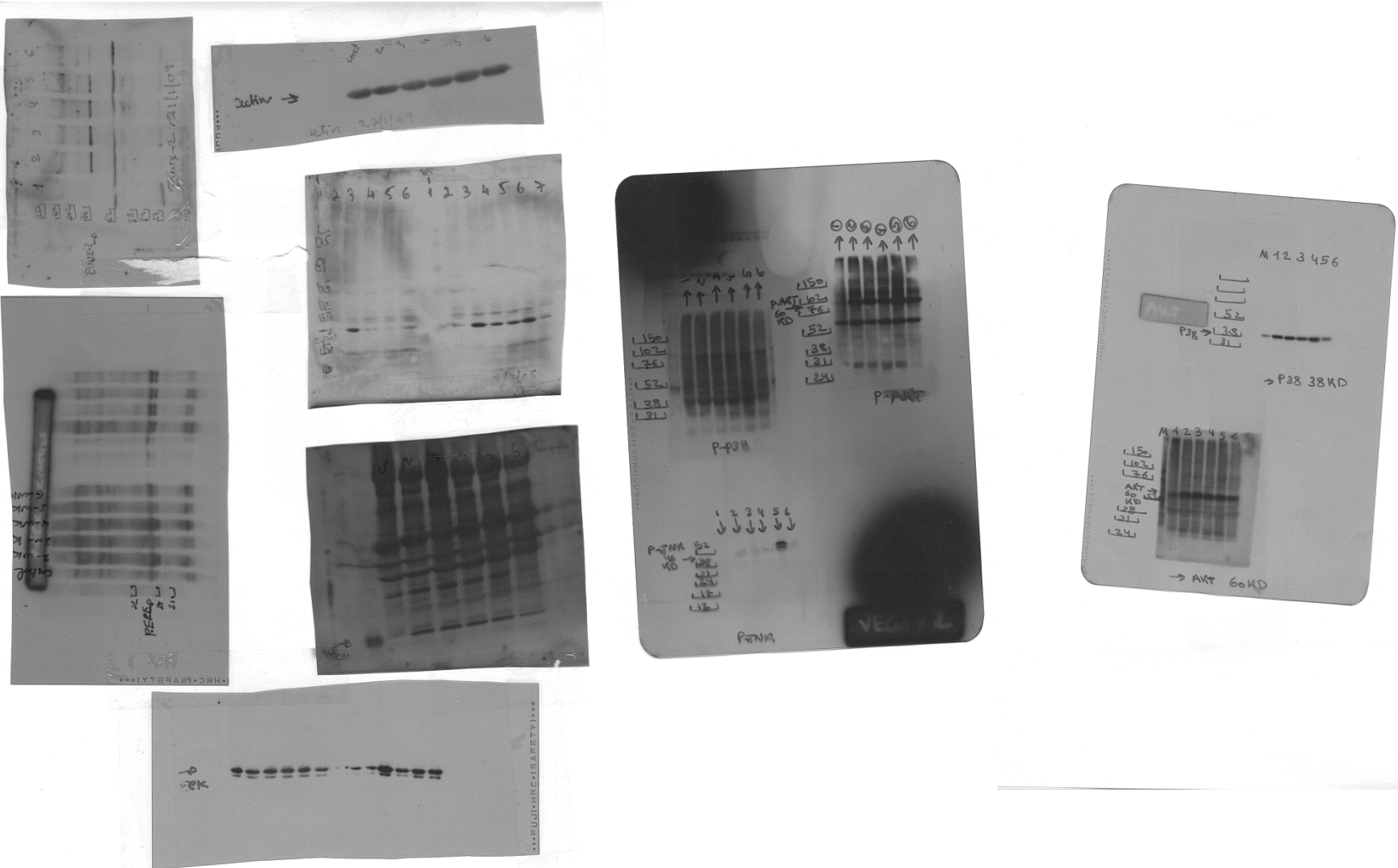

Supplement: S1 Fig — (PDF) [file pone.0129725.s001.pdf]
